# Rapid prediction of antibiotic resistance in *Enterobacter cloacae* complex using whole-genome and metagenomic sequencing

**DOI:** 10.1128/msystems.00584-25

**Published:** 2025-06-12

**Authors:** Fen Pan, Peng Han, Qianyue Wu, Wenqi Hou, Guanhua Rao, Zhan Ma, Wenhao Weng, Hong Zhang

**Affiliations:** 1 Department of Clinical Laboratory, Shanghai Children’s Hospital, School of Medicine, Shanghai Jiao Tong University 56694, Shanghai, China; 2 Institute of Pediatric Infection, Immunity, and Critical Care Medicine, Shanghai Jiao Tong University School of Medicine 56694, Shanghai, China; 3 College of Health Science and Technology, Shanghai Jiao Tong University School of Medicine 56694, Shanghai, China; 4 Genskey Medical Technology Co. Ltd. 637490 , Beijing, China; Chinese Academy of Sciences, Beijing, China

**Keywords:** metagenomic sequencing, machine learning, antimicrobial susceptibility testing, *Enterobacter cloacae *complex, whole-genome sequencing, antimicrobial resistance prediction

## Abstract

Clinical management and surveillance of the *Enterobacter cloacae* complex (ECC) face significant challenges due to inaccurate species identification and prolonged turnaround time for culture-based antimicrobial susceptibility testing (AST). To date, no studies have leveraged whole-genome sequencing (WGS) and metagenomic next-generation sequencing (mNGS) to develop a rapid AST prediction model for ECC. Here, a total of 1,054 ECC strain genomes with AST data were collected from a public database and a local hospital. The results of species identification between the average nucleotide identity (ANI)-based method on culture were compared, and machine learning was employed to identify resistance features for imipenem (IPM), meropenem (MEM), ciprofloxacin (CIP), levofloxacin (LEV), and trimethoprim-sulfamethoxazole (SXT). By referring to ANI-based species classification, culture-based methods showed a 74% misidentification rate for 1,054 ECC isolates. The antimicrobial resistance prediction model demonstrated good performance, with the area under the curve values of 91.25% (IPM), 89.69%, 88.17% (CIP), 91.01% (LEV), and 90.93% (SXT) respectively. Moreover, a combined WGS and mNGS approach was utilized and validated using 104 pediatric sputum specimens. Compared to culture-based AST, the overall accuracy of models exceeded 95%, especially achieving 100% for IPM and 98.80% for MEM, and the detection turnaround time was shortened by 69.64 h. Furthermore, it would enable early escalated therapy in 20.83% of cases, significantly improving patient management. This established WGS and mNGS-based AST prediction model addresses the limitations of traditional methods, offering a rapid, accurate, and clinically applicable tool for managing multidrug-resistant ECC infections.

**IMPORTANCE:**

The *Enterobacter cloacae* complex (ECC) poses a major challenge to clinical management due to difficulties in accurate species identification and the slow turnaround times of conventional culture-based antimicrobial susceptibility testing (AST). Current methods are often inefficient and prone to misidentification, leading to delayed or inappropriate treatment. This study introduces a novel approach that combines whole-genome sequencing (WGS) and metagenomic next-generation sequencing (mNGS) to develop a rapid and accurate AST prediction model for ECC. By leveraging machine learning to analyze WGS data from over 1,000 ECC isolates and validating the model with pediatric clinical specimens. The model achieved over 88% area under the curve accuracy for all antibiotics, demonstrated >95% accuracy in clinical validation, and reduced detection turnaround time by 69.64 h compared to traditional methods. The model has the potential to revolutionize ECC management by facilitating timely, targeted therapies and enhancing patient outcomes, especially in the context of multidrug-resistant infections.

## INTRODUCTION

The *Enterobacter cloacae* complex (ECC), including *E. cloacae*, *Enterobacter hormaechei*, *Enterobacter kobei*, *Enterobacter asburiae*, and *Enterobacter cancerogenus*, is an important cause of nosocomial infections such as pneumonia, urinary tract infections, and septicemia. The emergence of carbapenem-resistant ECC (CREC), as a member of the ESKAPE group (1), exhibiting multidrug-resistant (MDR) phenotypes, has become a growing public health concern due to the widespread use of antimicrobial agents, particularly carbapenems (2, 3). Traditional culture-based antimicrobial susceptibility testing (AST) is often time-consuming, typically requiring 48–72 h, which significantly delays targeted treatment and can negatively impact patient outcomes (4, 5). In the context of rapidly evolving antimicrobial resistance (AMR) and the alarming spread of CREC, there is a critical need for rapid, precise, and accurate AST methods to guide clinical decision-making.

Several novel rapid AST technologies, such as matrix-assisted laser desorption ionization-time of flight mass spectrometry (MALDI-TOF MS) and molecular-based genotypic methods, were explored (6, 7). Among these, next-generation sequencing (NGS) technologies, including whole-genome sequencing (WGS) and metagenomic NGS (mNGS), have shown great promise for predicting AMR (8, 9). mNGS, as a culture-independent and unbiased molecular technology, has been widely used in the detection and diagnosis of clinical infectious pathogens (10–12). Previous studies have demonstrated the high concordance of WGS or mNGS-based AST prediction with conventional culture-based AST results, particularly in MDR gram-negative *Klebsiella pneumoniae* (5, 13), *Escherichia coli* (14), *Pseudomonas aeruginosa* (15), and *Acinetobacter baumannii* (16). However, the performance of WGS or mNGS-based AST prediction for ECC is still unknown.

In this study, we integrated WGS and mNGS to predict antibiotic resistance in ECC. This combined approach, while previously applied to other pathogens, represents a novel contribution to ECC, a highly complex and MDR bacterial group. By exploring genotype-phenotype correlations, we developed a predictive model that outperforms conventional AST methods in terms of accuracy and clinical applicability. mNGS, as a culture-independent technology, allows for rapid, direct identification of resistance genes from clinical specimens, facilitating timely treatment recommendations. To assess the clinical utility of our model, we validated it using sputum samples from pediatric patients, highlighting its potential to improve diagnostic efficiency and optimize treatment outcomes in clinical practice.

## MATERIALS AND METHODS

### Collection of ECC genomes with AST data

**Fig 1:**
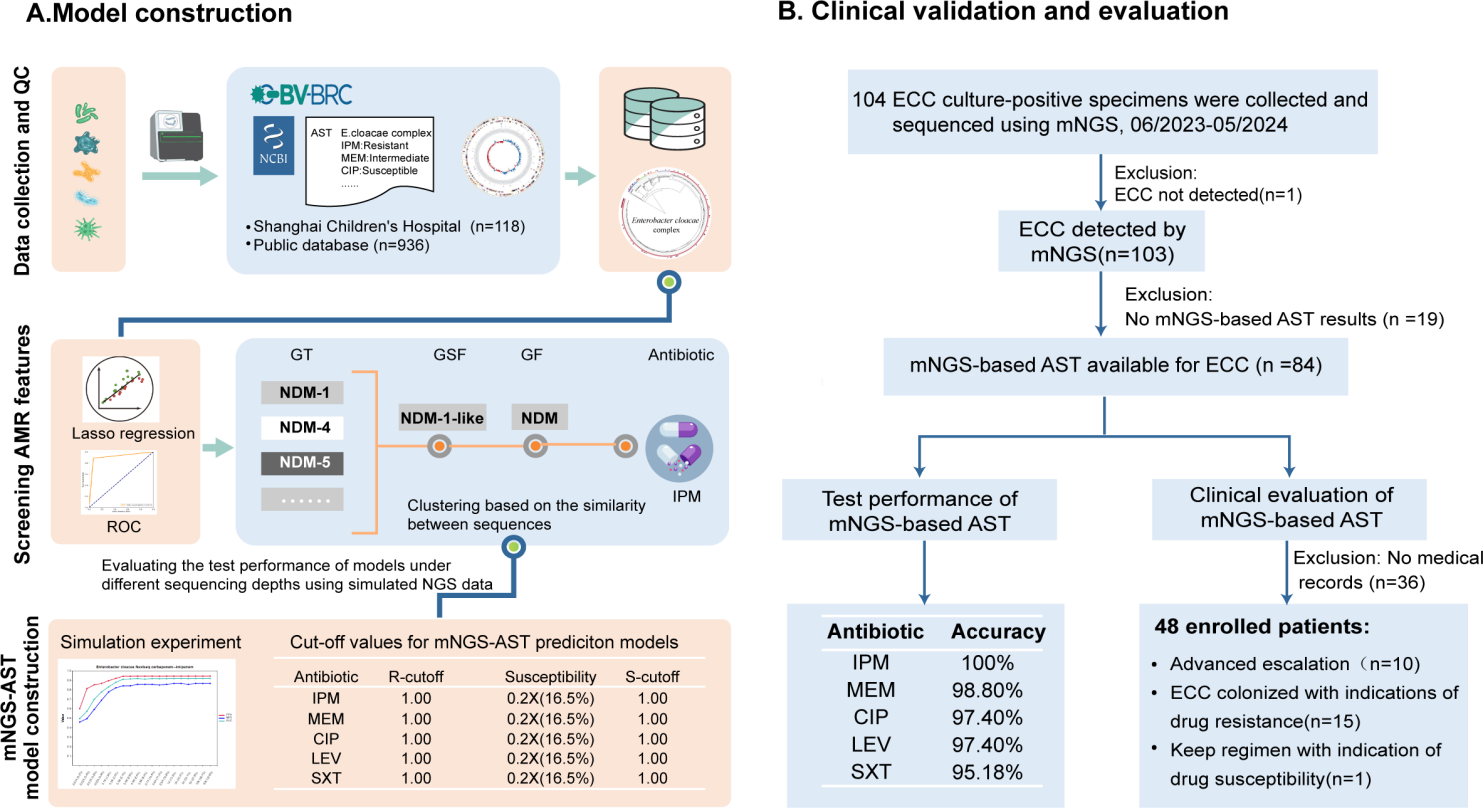
Study overview. The mNGS-AST prediction model was constructed for *Enterobacter cloacae* complex (ECC) and assessed by clinical specimens collected from pediatric patients. (A) Model construction: Whole-genome data of the ECC with antibiotic susceptibility testing results were collected from public databases (BV-BRC and NCBI NDARO) and Shanghai Children’s Hospital, followed by quality control to obtain a high-quality data set of genomes and AST results. Then, a lasso regression model was utilized to explore the relationship between genotype and phenotype and screen for key genomic features significantly associated with antibiotic resistance. Next, the mNGS-AST prediction model was applied, and the cutoffs were determined by simulation experiment of WGS data. (B) Clinical validation and evaluation: Sputums were collected from Shanghai Children’s Hospital to assess the model’s accuracy in predicting antibiotic susceptibility results. Meanwhile, assuming that the model’s predicted results were obtained by clinicians at that time, the intervention effect of clinical medication could be assessed for patients with complete antibiotic usage records. AMR, antimicrobial resistance; AST, antimicrobial susceptibility testing; CIP, ciprofloxacin; GF, gene family; GSF, gene subfamily; GT, gene type; IPM, imipenem; Lev, levofloxacin; MEM, meropenem; NDARO, National Database of Antibiotic Resistant Organisms; PATRIC, Pathosystems Resource Integration Center; SXT, trimethoprim-sulfamethoxazole.

On 24 November 2023, a total of 963 non-redundant whole-genome sequences, along with corresponding AST data for ECC, were retrieved from the BV-BRC and NCBI NDARO databases (Fig. 1A). After quality control, genomes with low-quality assembly or inconsistent AST data were excluded, yielding a final set of 936 genomes (854 from BV-BRC and 82 from NCBI NDARO). Additionally, 118 ECC strains were isolated from Shanghai Children’s Hospital (October 2020 to December 2023) and subjected to WGS. All 118 isolates were identified by MALDI-TOF MS using MALDI Biotyper (Bruker Daltonik GmbH, Bremen, Germany). The AST was conducted by Vitek2 automated susceptibility testing system (bioMérieux, Marcy l’Étoile, France) or by disk diffusion method. The breakpoints used for interpretation were recommended by the Clinical and Laboratory Standards Institute 2023 (Table S2). In total, 1,054 ECC strains were obtained, with a comparable distribution of resistant and sensitive strains (Table S1).

### Phylogenetic tree, average nucleotide identity (ANI), and multilocus sequence typing (MLST) analysis

To assess the diversity of the collected strains, single-copy core genes were identified for constructing the phylogenetic tree. The ANI and MLST analyses were performed to establish taxonomic information and genomic sequence types. Reference genomes for various species within the ECC were downloaded from the NCBI genome database, and ANI analysis was conducted using FastANI software (17). Strains with ANI ≥ 95% were classified as the same species. For strains with ANI values > 95% against multiple reference genomes, the species with the highest ANI value was assigned. Seven housekeeping genes (*dnaA*, *fusA*, *gyrB*, *leuS*, *pyrG*, *rplB*, and *rpoB*) were obtained from the pubMLST database (https://pubmlst.org), and the genomes of each strain were aligned to these genes using BLASTN (version 2.13.0+) to determine the MLST sequence types (18). The phylogenetic tree was visualized using iTOL (19), with annotations for species classification and AST data.

### Identification of key AMR features using Lasso regression

We employed two approaches to identify AMR features for ECC and evaluate the performance of the AMR prediction model, as described in previous studies (14, 16). First, we aligned genome contig sequences against genes listed in the CARD database (20) to identify candidate AMR features. Second, predicted open reading frames from the genomes were used to obtain additional candidate resistance features. The Lasso regression model was then applied to assess the association between candidate resistance features and AST phenotypes. Key resistance features for imipenem (IPM), meropenem (MEM), ciprofloxacin (CIP), levofloxacin (LEV), and trimethoprim-sulfamethoxazole (SXT) were identified based on model performance.

Given that the AMR prediction model was designed for the ECC complex level, we further examined the impact of different species within ECC on model performance. For CIP and LEV, where gene mutations are the primary resistance determinants, we analyzed the homology of *gyrA*, *gyrB*, *parC*, and *parE* gene sequences across all ECC strains. Susceptibility-type sequences from seven major species were selected as references to evaluate their impact on the AMR prediction model. For example, we performed clustering analysis on the *gyrA* sequences of 1,054 strains, selecting non-redundant susceptibility-type sequences from the seven species with the highest strain counts as reference sequences (Fig. S1). The amino acid polymorphisms at locus 83 of the *gyrA* gene, which are significantly associated with quinolone resistance, were further analyzed (Table S5).

### mNGS-AST prediction model optimization and threshold setting

The mNGS-AST prediction model previously developed (13, 16) was applied to predict the antibiotic susceptibility of ECC based on the key AMR features identified using clinical mNGS data. In brief, when an AMR feature was detected, the result was reported as “resistant” (R). In the absence of detectable AMR features, the sequencing depth was assessed. If the depth was sufficient, “susceptible” (S) was reported. Otherwise, no clear AST conclusion could be provided. The minimum sequencing depth required for reporting susceptibility was evaluated through simulation experiments. Specifically, the ART software (v2.5.8) was used to simulate WGS reads data with different amounts of sequencing data, such as 0.05×, 0.1×, 0.2×, 0.3×, 0.4×, 0.5×, 0.6×, 0.7×, 0.8×, 0.9×, 1×, 2×, 3×, 5×, 10×, 30×, in order to evaluate the performance of the model at different sequencing depths. When the performance of the model reaches stability, it indicates that this drug-resistant feature can be stably detected. Then, the lowest amount of sequencing data corresponding to the stable performance of the model can be defined as the minimum sequencing depth for reporting sensitivity. Next, for CIP and LEV, mainly characterized by variant-type AMR features, the method for reporting susceptibility was optimized by assessing the coverage of key variant resistance sites in genes such as *gyrA* and *parC,* rather than evaluating the coverage of the genome. This optimization increased the proportion of reportable cases. The threshold for reporting resistance and the minimum sequencing depth required for reporting susceptibility were evaluated and established through simulated sequencing data (Table S7).

### Retrospective evaluation of model performance using clinical specimens

From October 2022 to June 2024, 104 clinical specimens of suspected respiratory tract infection patients with positive ECC culture were collected at Shanghai Children’s Hospital, including 103 sputum samples and 1 bronchoalveolar lavage fluid sample. All specimens were sent to Genskey Medical Laboratory for mNGS sequencing, and the mNGS-AST prediction model was applied to predict the antibiotic susceptibility of ECC. The model’s predictions were compared with traditional culture-based AST results. The effective report ratio, positive predictive value (PPV), negative predictive value (NPV), and accuracy were calculated to assess the model’s performance.

Furthermore, 48 patients with complete medical records of antibiotic use were selected for a review. Assuming that clinicians had access to the AST prediction results promptly, the impact of the model on clinical treatment was evaluated (Fig. 1B). In detail, in real-world clinical management, specimens were collected on D0 for bacterial culture and clinical mNGS. Clinicians could adjust treatment on D1 using mNGS results and further from D2 to D7 with culture results and patient response. To evaluate the model’s clinical benefits, a simulated experiment assumed mNGS-based AST results were available on D1. Physicians provided antibacterial therapy recommendations (de-escalation, escalation, and maintenance) based on mNGS-based or culture-based AST. Inconsistent cases were re-evaluated. In simulation, we assessed the antibacterial resistance of confirmed pathogens on D1 per each AST method. For culture-positive patients, we compared physician recommendations, and consistent ones indicated mNGS-based AST’s positive impact due to earlier adjustments.

## RESULTS

### Population diversity

**Fig 2:**
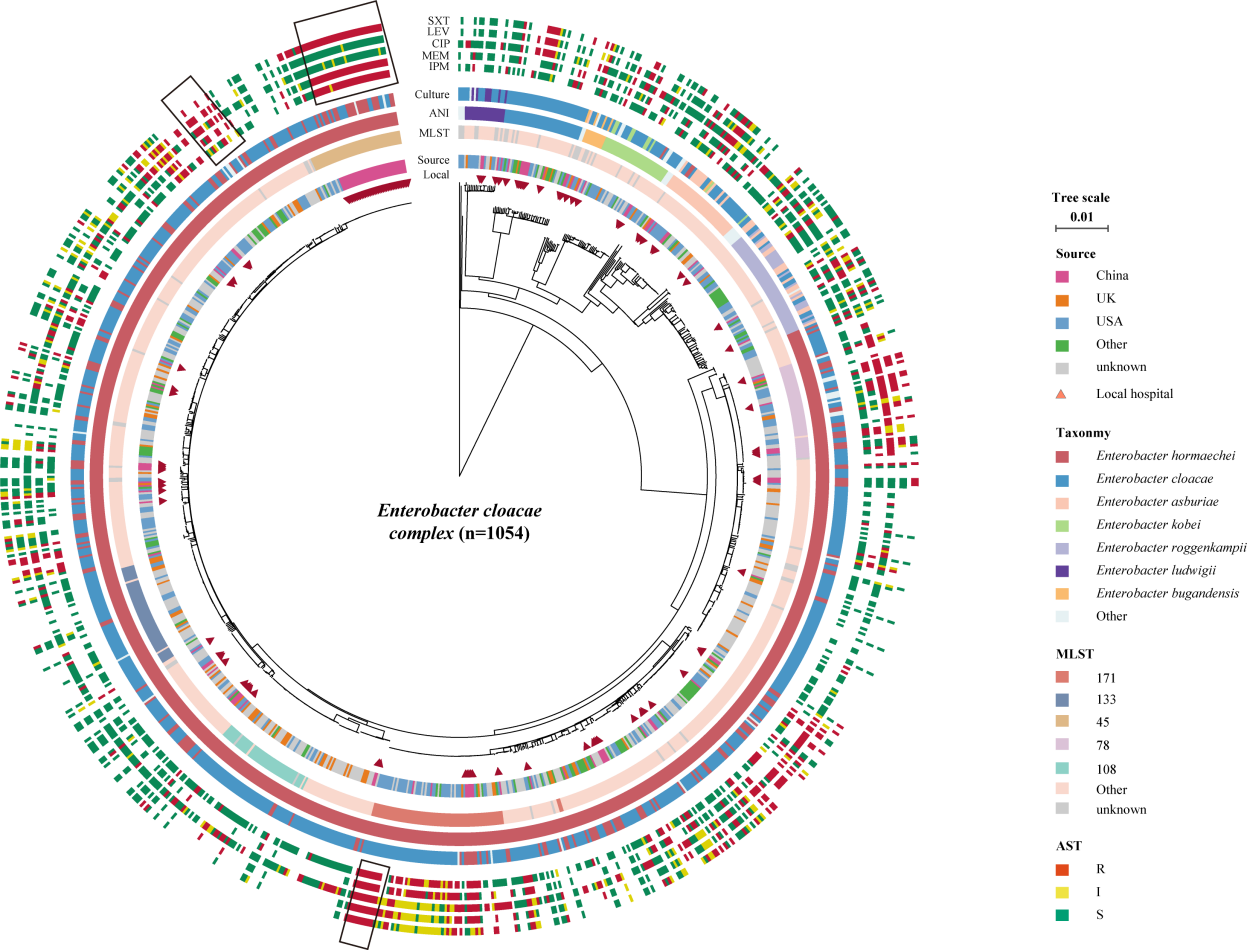
Phylogenetic tree with annotations of geographic source, species classification, and AST conclusion for 1,054 *Enterobacter cloacae* complex (ECC) isolates. The maximum likelihood phylogenetic tree was constructed based on the core genes and plotted for ECC using iTOL (https://itol.embl.de). Circles from the inside out represent the geographical origin, the species classification information (including traditional culture-based identification and ANI-based classification), and the AST results of IPM/MEM/CIP/LEV/SXT. I, intermediate; R, resistant; S, susceptible. The innermost red triangle indicates the source of the strains, which were collected from Shanghai Children’s Hospital.

A total of 1,054 ECC strains with AST data were collected. Of these, 936 strains were sourced from public databases representing regions worldwide, including the United States, China, and the United Kingdom, while the remaining 118 strains were obtained from Shanghai Children’s Hospital. The number of strains with available susceptibility data for IPM, MEM, CIP, LEV, and SXT ranged from 408 to 679 (Tables S1 and S2). Phylogenetic analysis revealed substantial genetic polymorphism among the ECC strains. Notably, resistance phenotypes correlated with certain phylogenetic subgroups, with some displaying multidrug resistance or carbapenem resistance. However, a strain’s antimicrobial susceptibility phenotype was not strictly determined by its phylogenetic position (Fig. 2). ANI analysis showed strong concordance between species classification derived from ANI and phylogenetic tree results, but substantial discrepancies (up to 74%) were observed when compared with traditional culture-based identification (Table S2). Based on ANI classification, the most prevalent ECC species were *E. hormaechei* (80.93%, 853/1,054), *Enterobacter roggenkampii* (4.84%, 51/1,054), and *E. cloacae* (3.51%, 37/1,054).

### Evaluation of model performance using lasso regression and screening key AMR features

**Fig 3:**
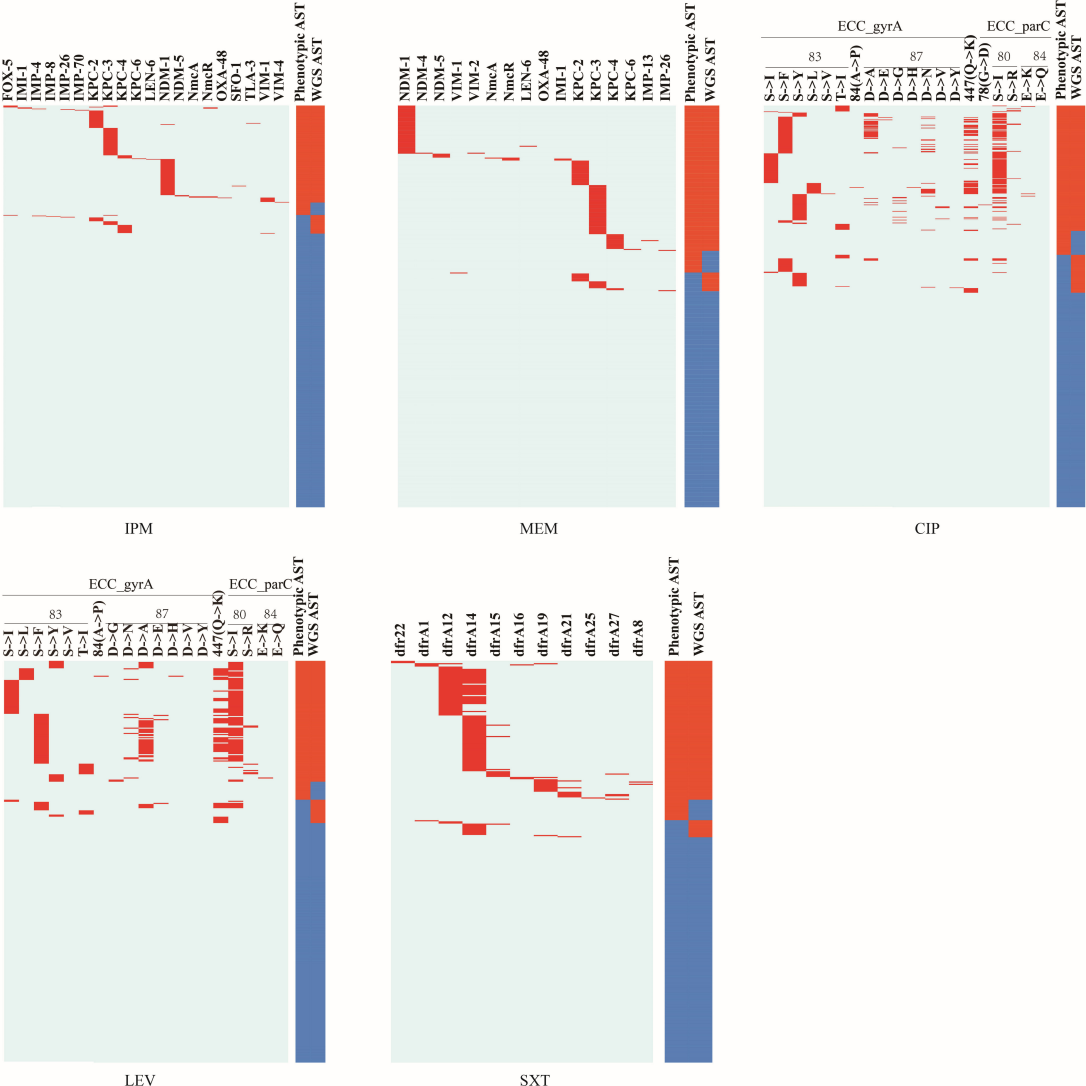
Using the Lasso regression model to screen AMR features. Heatmap of key antimicrobial resistance (AMR) features for IPM, MEM, CIP, LEV, and SXT. The presence of AMR features in each strain is shown in the left panel. The results of routine AST and WGS-AST are shown in the right panel.

The area under the curve (AUC) values for the models predicting AMR in ECC strains were as follows: 91.25% for IPM, 89.69% for MEM, 88.17% for CIP, 91.01% for LEV, and 90.93% for SXT. The number of resistance genes varied from 1 to 11, while the number of resistance features ranged from 6 to 20 (Table 1; Table S4). For IPM, MEM, and SXT, the presence or absence of specific resistance genes was the primary determinant. The key resistance genes for IPM and MEM were blaKPC, blaNDM, blaVIM, and NmcR, while the dfr gene family was crucial for SXT. For CIP and LEV, gene mutations, particularly at the 80th amino acid position of *parC* and the 83rd amino acid position of *gyrA*, were the primary resistance features (Fig. 3). When applied to individual species within the ECC complex, the models showed comparable performance for IPM, MEM, and SXT, which were primarily based on gene presence or absence (GPA), and for CIP and LEV, which relied on mutation features (Table S3).

**TABLE 1:** Performance of the Lasso regression classifier to predict susceptibility or resistance to five antibiotics for *Enterobacter cloacae* complex^*a*^

Antibiotic	No. of true resistance	No. of false resistance	No. of true susceptibility	No. of false susceptibility	Sensitivity (%)	Specificity (%)	PPV (%)	NPV (%)	Accuracy (%)	AUC	Type of AMR features	No. of markers	No. of genes
IPM	156	30	440	20	88.64	93.62	83.87	95.65	92.26	0.913	GPA	20	11
MEM	148	19	220	22	87.06	92.05	88.62	90.91	89.98	0.897	GPA	16	8
CIP	215	65	368	41	83.98	84.99	76.79	89.98	84.62	0.882	GPA, SNP	7	2
LEV	114	22	226	17	87.02	91.13	83.82	93	89.71	0.91	GPA, SNP	6	2
SXT	122	15	198	18	87.14	92.96	89.05	91.67	90.65	0.909	GPA	11	1

^*a*^ True resistance, phenotypical AST is resistant and genotypic resistance is predicted. False resistance, phenotypical AST is susceptible and genotypic resistance is predicted. True susceptibility, phenotypical AST is susceptible and genotypic susceptibility is predicted. False susceptibility, phenotypical AST is resistant and genotypic susceptibility is predicted. AMR, antimicrobial resistance; AUC, area under curve; CIP, ciprofloxacin; GPA, gene presence or absence; IPM, imipenem; LEV, levofloxacin; MEM, meropenem; No. of markers, the number of features determined when AUG value is higher or cross-validation error is small; SNP, single nucleotide polymorphisms; SXT, trimethoprim-sulfamethoxazole.

Further analysis of polymorphisms in the *gyrA* gene across species within the ECC complex showed that the homology of the *gyrA* gene ranged from 93% to 95.9% among seven main species. Most species encoded serine (S) at the 83rd amino acid position of *gyrA*, while *E. kobei* and *E. roggenkampii* encoded threonine (T) (Table S5). Adding susceptibility-type sequences for *gyrA*, *parC, gyrB*, and *parE* genes from seven species did not significantly affect model performance compared to using *E. hormaechei* sequences alone (Table S6).

### Validation of model performance using clinical specimens

In a validation study involving 104 clinical samples, mNGS detected ECC strains with genomic coverage ranging from 0.05% to 89.39% (Table S8). The proportion of samples yielding valid antimicrobial susceptibility predictions for the five antibiotics was 80.76% (84/104). The effective reporting ratios for each resistance model were as follows: IPM 79.81% (83/104), MEM 79.81% (83/104), CIP 74.04% (77/104), LEV 74.04% (77/104), and SXT 79.81% (83/104). The accuracy of the mNGS-AST prediction models for the five antibiotics exceeded 95%, with accuracies of 100% for IPM, 98.80% for MEM, 97.40% for CIP, 97.40% for LEV, and 95.18% for SXT. PPVs for all models were greater than 90%. The NPVs were 100% for IPM, 98.08% for MEM, 97.26% for CIP, 97.26% for LEV, and 97.92% for SXT (Table 2). Compared to conventional culture-based AST, the mNGS-AST prediction model reduced the average turnaround time by 69.64 h (17.95 ± 0.15 h vs 87.59 ± 18.03 h) (Table S9).

**TABLE 2:** Performance of read-based mNGS-AST model to predict susceptibility or resistance to different antibiotics of *Enterobacter cloacae* complex in 104 clinical specimens^*a*^

Antibiotic	Strain (R|I|S)	No. of true resistance	No. of VME	No. of true susceptibility	No. of ME	Accuracy (%)	PPV (%)	NPV (%)	Predictable rate (%)		
									Overall	In R samples	In S samples
IPM	38|0|66	31	0	52	0	100	100	100	37.35 (31/83)	81.58 (31/38)	78.79 (52/66)
MEM	37|2|65	31	1	51	0	98.8	100	98.08	37.35 (31/83)	82.05 (32/39)	78.46 (51/65)
CIP	8|1|95	4	2	71	0	97.4	100	97.26	5.19 (4/77)	66.67 (6/9)	74.74 (71/95)
LEV	8|1|95	4	2	71	0	97.4	100	97.26	5.19 (4/77)	66.67 (6/9)	74.74 (71/95)
SXT	37|0|67	32	1	47	3	95.18	91.43	97.92	42.17 (35/83)	89.19 (33/37)	74.63 (50/67)

^*a*^ VME, very major error, phenotypic AST indicates resistance while genotypic AST indicates susceptibility; ME, major error, phenotypic AST indicates susceptibility while genotypic AST indicates resistance. PPV, positive predictive value, calculated as true resistance/(true resistance + ME); NPV, negative predictive value, calculated as true susceptibility/(true susceptibility + VME). Predictable rate, the percentage of predicSupplementary Table Samples to all samples with clear AST conclusion. In_R_samples, the predictable rate in resistant samples; In_S_samples, the predictable rate in susceptible samples. IPM, imipenem; MEM, meropenem; CIP, ciprofloxacin; LEV, levofloxacin; SXT, trimethoprim-sulfamethoxazole.

**Fig 4:**
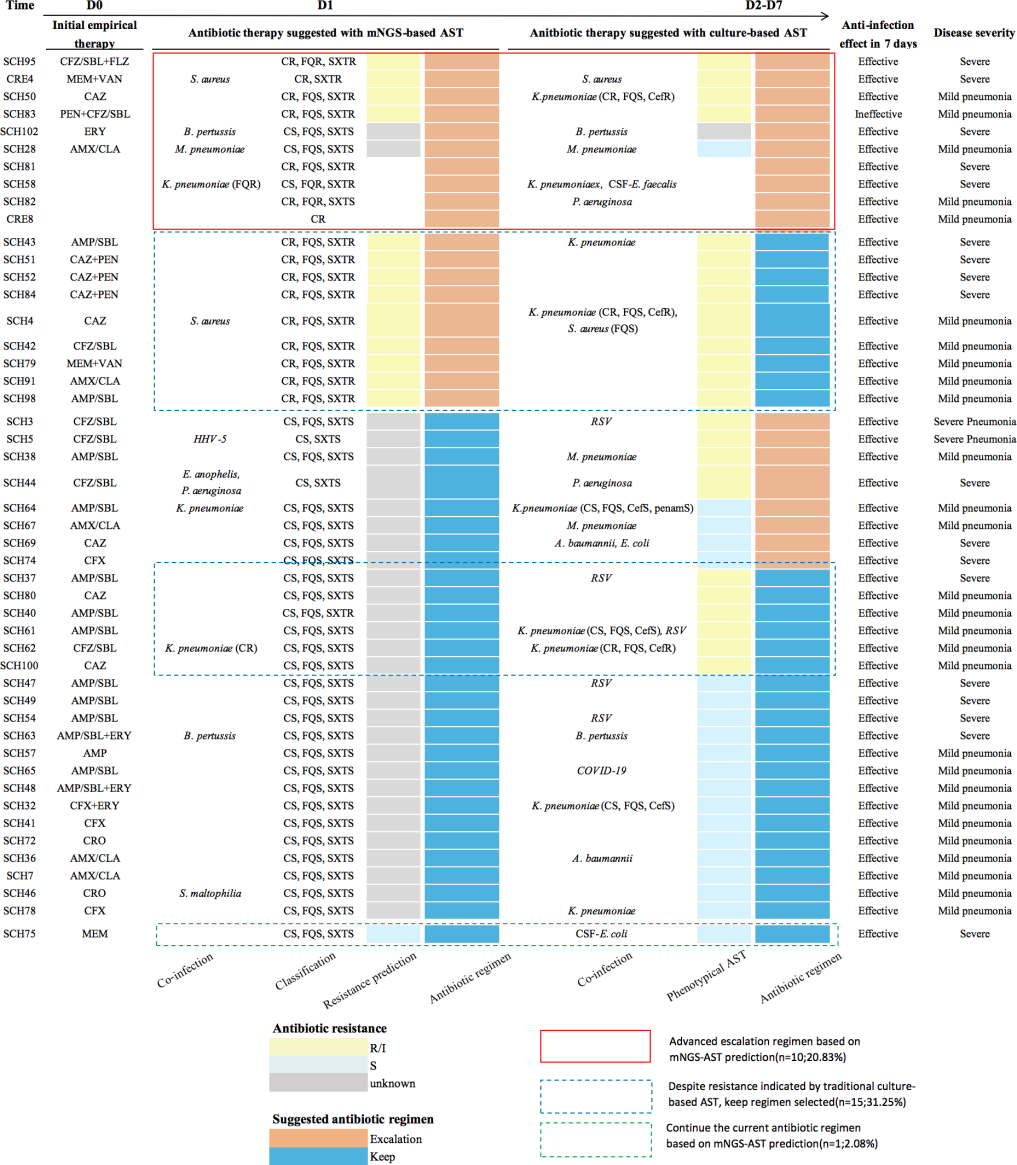
Evaluation of the intervention effect of antibiotic use. Schematic diagram of a review of 48 cases of suspected bacterial infections. The first column is the sample ID. The second column is the initial antibiotic treatment on D0. The middle section shows the assessment of the clinician’s recommended antibiotic therapy suggested with mNGS and mNGS-AST assay. The right section is the assessment of the clinician’s recommended antibiotic therapy suggested without mNGS and mNGS-AST assay. AMP, ampicillin; AMP/SBL, ampicillin-sulbactam; AMX/CLA, amoxicillin-clavulanic acid; CAZ, ceftazidime; CFX, cefuroxime; CR/CS, carbapenem resistant/susceptible; CRO, ceftriaxone; De-escalation, change to a more narrow-spectrum antibiotic agent; ERY, erythromycin; Escalation, change to a broader antibiotic regimen or add other antibiotic regimens; FQR/FQS, fluoroquinolone resistant/susceptible; FZ/SBL, cefoperazone-sulbactam; Keep, continue the current antibiotic regimen; MEM, meropenem; PEN, penicillin; R/I, resistant or intermediate; S, susceptible; SXTR/S, trimethoprim-sulfamethoxazole resistant/susceptible; VAN, vancomycin..

For 48 patients with complete medication history, the impact of mNGS-based pathogen detection and AST prediction on clinical treatment decisions was evaluated. Assuming physicians received pathogen and antimicrobial susceptibility results on Day 0 (D0), approximately 20.83% (10/48) of patients could have benefited from earlier escalation of therapy. However, 31.25% (15/48) of patients, initially considered colonized with ECC by culture-based AST, received effective treatment based on the original antimicrobial regimen initiated on D0 (Fig. 4).

## DISCUSSION

In this study, we evaluated the effectiveness of the mNGS-based AST prediction method for ECC, with a focus on predicting its susceptibility and resistance to IPM, MEM, LEV, CIP, and SXT. We first collected a comprehensive set of ECC genomes with AST data from public databases and a local hospital. A machine learning model was then employed to identify key resistance features associated with resistance phenotypes. These selected features were incorporated into the mNGS-based AST prediction model, which was subsequently validated using clinical specimens. The results demonstrated that the model achieved over 95% accuracy and could predict bacterial antibiotic susceptibility or resistance from clinical specimens within 24 h, providing a powerful tool for guiding rapid and precise antibiotic therapy.

The ECC contains multiple species, and there are differences in drug resistance and virulence among different species (21). To construct an antibiotic resistance prediction model for ECC, it is first necessary to accurately classify ECC species and evaluate the model performance of different species. Traditional phenotypic methods like API 20E and Vitek 2, as well as mass spectrometry, often fail to accurately differentiate species within the ECC, except for *E. cloacae* and *E. asburiae*. This limitation hampers precise identification of clinically relevant species such as *E. hormaechei* and *E. kobei* (3, 22). WGS-based species classification methods, especially ANI and dDDH, are widely used for precise classification and identification of species. Here, we calculated the ANI value based on WGS to identify all ECC strains from public databases and a local hospital. The primary species identified through WGS was *E. hormaechei* (80.93%), consistent with previous studies (21, 23), whereas traditional culture-based methods predominantly identified *E. cloacae* (71.73%). The concordance between WGS-based and culture-based species identification was only 26%, highlighting significant discrepancies between these methodologies. According to WGS-based species classification results, we next separately constructed models for the ECC and each species within ECC and found that the model performance at the complex level was comparable to that at the species level (AUC: 88.17–91.25%). This indicates that the previous strategy for constructing drug resistance models is not only applicable at the species level (4, 13–16), but also may be directly applied at the complex level. Moreover, for antibiotics such as CIP and LEV, which exhibit variable resistance characteristics, we examined whether increasing the number of wild-type templates of ARG could improve the accuracy of the model. Although the similarity of *gyrA* sequences among different species within ECC may be low, even below 95% (Table S5), integrating more wild-type templates did not significantly improve model performance, but it significantly increased the identification accuracy of the mutation sites of drug resistance genes (Table S5).

To enable mNGS-based AST in clinical specimens, we utilized a large data set of ECC-resistant strains and applied machine learning methods to identify key resistance features or markers, which were then integrated into the predictive model. This strategy enables direct and accurate prediction of ECC resistance from mNGS data, offering significant time advantages compared to conventional culture-based AST. In contrast to predictions based solely on publicly available AMR genes, our approach provides enhanced predictive precision (24). The model achieved over 95% accuracy in predicting resistance in clinical specimens, particularly for carbapenem antibiotics, with only one case of false susceptibility. Notably, the mNGS-AST model was developed at the ECC complex level rather than at the individual species level. This design choice aligns with clinical practice, where therapeutic decisions are commonly based on complex-level identification, and it provides greater clinical relevance without compromising diagnostic accuracy. To assess clinical applicability, we simulated a scenario in which clinicians received the mNGS-AST report within 1–2 days post-testing. The simulation showed that 31.25% (15/48) of patients had mNGS-detected resistance to their initial empirical antibiotic treatment (IEAT), but no modifications were made to their therapy. These findings underscore the importance of considering potential ECC colonization, especially in pediatric patients with suspected respiratory infections, even in the presence of resistant strains detected by mNGS. A comprehensive evaluation of IEAT efficacy, AST results of detected pathogens, and co-infections is essential to inform clinical decision-making.

Despite the promising results, there were some limitations to this study. First, due to the high proportion of human-derived sequences in clinical specimens, the genomic coverage of pathogenic bacteria measured under conventional sequencing data of 20M sequences is often less than 100%. Therefore, although ECC was detected by mNGS, 19.25% (84/104) of all samples could not be clearly predicted as sensitivity when the corresponding resistance characteristics were not detected. Second, two out of nine resistant clinical specimens were incorrectly predicted as susceptible to CIP and LEV, indicating that some potential resistance features may not have been identified from our collected ECC strains (25). This suggests that further investigation into quinolone resistance mechanisms is necessary, and we need to collect more resistant ECC strains in the future to update and optimize the model. Third, as the isolates were obtained solely from a paediatric hospital, the sample size and diversity are inherently limited. Broader validation using clinical samples from general hospitals is warranted to assess model robustness and extend its clinical relevance.

In conclusion, we have introduced an mNGS-based method that directly predicts antibiotic susceptibility for ECC from clinical specimens. This approach provides a timely and effective tool for guiding precise clinical prescribing, offering a significant advancement in the rapid management of ECC-related infections. Our findings highlight the potential of mNGS-based diagnostic tools to revolutionize clinical decision-making and improve patient outcomes, especially in the face of rising AMR.

## Data Availability

The prediction pipeline was developed mainly using Perl, R, and published sequence alignment software including ncbi-blast-2.9.0+ and minimap2 (version2.17). The code of prediction pipeline and shotgun metagenomic sequencing data are available from the corresponding author upon request. The sequencing data that support the findings of this study have been deposited into CNGB Sequence Archive (CNSA) with the accession number CNP0007461.

## SUPPLEMENTAL MATERIAL

Fig. S1 msystems.00584-25-s0001.docx Cluster analysis of nonredundant *gyrA* sequences with resistant phenotype distribution*.*

Supplemental tables msystems.00584-25-s0002.xlsx Tables S1 to S9.

ASM does not own the copyrights to Supplemental Material that may be linked to, or accessed through, an article. The authors have granted ASM a non-exclusive, world-wide license to publish the Supplemental Material files. Please contact the corresponding author directly for reuse.
