# Supplementary material for: Rapid prediction of antibiotic resistance in Enterobacter cloacae complex using whole-genome and metagenomic sequencing
Source: mSystems. 2025 Jun 12;10(7):e00584-25. doi: 10.1128/msystems.00584-25 (PMC12282082; doi:10.1128/msystems.00584-25)
Supplement: Fig. S1 — Cluster analysis of nonredundant gyrA sequences with resistant phenotype distribution. [file msystems.00584-25-s0001.docx]

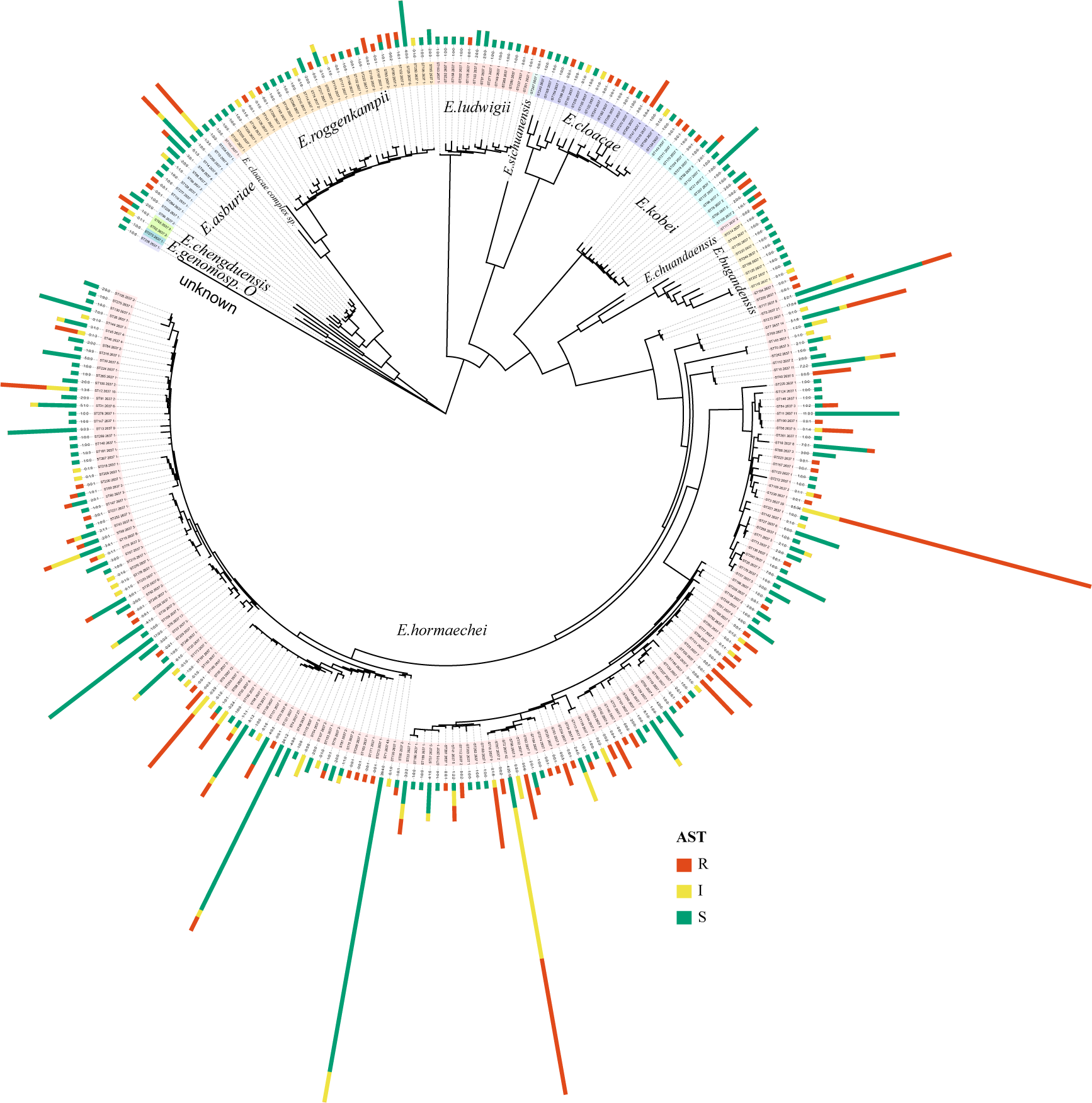


**Fig. S1 Cluster analysis of Non-redundant *gyr*A sequences with resistant phenotype distribution.**The *gyr*A sequences were extracted from 1054 Enterobacter cloacae complex genome sequences, and cluster analysis was performed after deduplication. The outermost circle represents antibiotic resistance phenotype. R, resistant; I, intermediate; S, susceptible.
